# Pre-treatment or Post-treatment of Human Glioma Cells With BIX01294, the Inhibitor of Histone Methyltransferase G9a, Sensitizes Cells to Temozolomide

**DOI:** 10.3389/fphar.2018.01271

**Published:** 2018-11-02

**Authors:** Iwona Anna Ciechomska, Marta Patrycja Marciniak, Judyta Jackl, Bozena Kaminska

**Affiliations:** Laboratory of Molecular Neurobiology, Neurobiology Center, Nencki Institute of Experimental Biology, Warsaw, Poland

**Keywords:** BIX01294, histone methyltransferase G9a, glioma, temozolomide, sensitization therapy

## Abstract

Glioblastoma (GBM) is a malignant, primary brain tumor, highly resistant to conventional therapies. Temozolomide (TMZ) is a first line therapeutic agent in GBM patients, however, survival of such patients is poor. High level of DNA repair protein, O^6^-methylguanine-DNA-methyltransferase (MGMT) and occurrence of glioma stem-like cells contribute to GBM resistance to the drug. Here, we explored a possibility of epigenetic reprograming of glioma cells to increase sensitivity to TMZ and restore apoptosis competence. We combined TMZ treatment with BIX01294, an inhibitor of histone methyltransferase G9a, known to be involved in cancerogenesis. Two treatment combinations were tested: BIX01294 was administered to human LN18 and U251 glioma cell cultures 48 h before TMZ or 48 h after TMZ treatment. Despite their different status of the *MGMT* gene promoter, there was no correlation with the response to TMZ. The analyses of cell viability, appearance of apoptotic alterations in morphology of cells and nuclei, and markers of apoptosis, such as levels of cleaved caspase 3, caspase 7 and PARP, revealed that both pre-treatment and post-treatment with BIX01294 sensitize glioma cells to TMZ. The additive effect was stronger in LN18 cells. Moreover, BIX01294 enhanced the cytotoxic effect of TMZ on glioma stem-like cells, although it was not associated with modulation of the pluripotency markers (*NANOG, SOX2, CD133*) expression or methylation of *NANOG* and *SOX2* gene promoters. Accordingly, knockdown of methyltransferase G9a augments TMZ-induced cell death in LN18 cells. We found the significant increases of the LC3-II levels in LN18 cells treated with BIX01294 alone and with drug combination that suggests involvement of autophagy in enhancement of anti-tumor effect of TMZ. Treatment with BIX01294 did not affect methylation of the *MGMT* gene promoter. Altogether, our results suggest that G9a is a potential therapeutic target in malignant glioma and the treatment with the G9a inhibitor reprograms glioma cells and glioma stem-like cells to increase sensitivity to TMZ and restore apoptosis competence.

## Introduction

Glioblastoma (GBM, WHO grade IV glioma) represents the largest group of brain tumors that remain incurable despite aggressive treatments (Jemal et al., 2005; Stupp et al., 2009). The current standard therapy for patients with newly diagnosed GBM consists of surgical resection, followed by radio- and chemotherapy. Temozolomide (TMZ) is the first therapeutic agent approved for the treatment of malignant gliomas, and when combined with radiotherapy improves both median survival and 5-year overall survival (Stupp et al., 2005, 2009). Despite those efforts, the prognosis of GBM patients remains poor and a median survival is 15 months after initial diagnosis. TMZ exerts cytotoxicity mainly by methylating guanine residues in DNA at O^6^ position and inducing DNA damage (Kaina et al., 1997; D’Atri et al., 1998), therefore it displays the highest efficacy against tumors lacking the DNA repair protein, O^6^-methylguanine-DNA-methyltransferase (MGMT). The lack of MGMT expression due to methylation of the *MGMT* gene promoter is prognostic for better outcome after TMZ chemotherapy (Hegi et al., 2008). One of the obstacle in GBM therapy is extensive heterogeneity at cellular and molecular levels. GBMs contain a rare population of glioma stem-like cells (GSCs), multipotent cells, capable of self-renewal and resistant to therapies. Residual GSCs can survive oncological therapies and then give rise to tumor recurrence (Reya et al., 2001; Bao et al., 2006; Chen et al., 2012).

One of the emerging therapeutic approaches is inducing differentiation of GSCs and epigenetic enzymes inhibitors, which can induce epigenetic reprogramming, are envisioned as potential anti-tumor therapeutics. Histone lysine methyltranferase G9a (EHMT2) catalyzes histone H3 dimethylation at lysine 9 (H3K9me2), the epigenetic mark that is strongly associated with gene repression (Tachibana et al., 2001; Shinkai and Tachibana, 2011). G9a has been found upregulated in many cancers, including brain tumors, and its overexpression has been associated with poor prognosis and a more aggressive phenotype of cancer (Casciello et al., 2015). G9a expression influences cancer cell motility and invasion by altering histone H3K9 methylation levels (Dong et al., 2012). Inhibition of G9a leads to the re-expression of cell adhesion molecules and reduction of motility and metastasis in aggressive lung and breast cancers *in vivo* (Chen et al., 2010; Dong et al., 2012). Elevated G9a levels are correlated with increased methylation leading to repression of tumor suppressor genes, i.e., the tumor suppressor TP53 (Huang et al., 2010). G9a depletion inhibits cell proliferation and induces cell differentiation (Ling et al., 2012; Yang et al., 2012; Culmes et al., 2013; Li et al., 2014). Therefore, there is a rationale to target G9a in cancer. BIX01294 (adiazepin-quinazolin-amine derivative), the most efficient inhibitor of G9a, transiently reduces methylation of H3K9 and H3K27 in different cancer cells, including glioma cells (Kubicek et al., 2007; Maleszewska et al., 2014; Ciechomska et al., 2016). At higher concentrations, BIX01294 impairs viability of rat and human glioma cells, which results in apoptosis (Maleszewska et al., 2014; Ciechomska et al., 2016). Interestingly, various studies have shown that G9a inhibition leads to autophagy induction (Artal-Martinez de Narvajas et al., 2013; Ciechomska et al., 2016). Autophagy is a dynamic recycling process associated with formation of autophagic vacuoles (Klionsky, 2007). Although autophagy is thought to be predominantly a cell-survival mechanism, some evidence points toward its role in cell death (Nikoletopoulou et al., 2013).

An emerging anti-tumor strategy is combining epigenetic enzyme inhibitors with conventional therapeutics. Different inhibitors of HDACs (histone deacetyltransferases) enhance radiation and TMZ responses *in vitro* and *in vivo* in various cancer cells, including glioma cells (Camphausen et al., 2005; Chinnaiyan et al., 2008; Van Nifterik et al., 2012). In the present study we sought to examine whether BIX01294, a specific inhibitor of G9a, could sensitize human glioma cells to TMZ. We hypothesized that G9a will affect the cancer stem cell capacity and restore glioma cell competence to undergo apoptosis in a response to TMZ. To test different schemes BIX01294 was administrated before (pre-treatment) or after adding TMZ (post-treatment). We used two glioma cell lines with unmethylated and methylated the *MGMT* gene promoter. Our results show that pharmacological (BIX01294) or genetic blockers of G9a (siRNA) restores cell capacity to undergo apoptotic death upon TMZ treatment, although without influence on the expression of stemness markers.

## Materials and Methods

### Reagents and Antibodies

Reagent and antibody sources were as follows: BIX01294 trihydrochloride hydrate, DAPI (4′,6-diamidino-2-phenylindole dihydrochloride), MTT (3-(4,5-dimethylthiazol-2-yl)-2,5-diphenyltetrazolium bromide), TMZ, anti-LC3 and anti-β-Actin-peroxidase conjugated antibody (Sigma-Aldrich, Munich, Germany), anti-cleaved caspase 3, anti-cleaved caspase 7, anti-cleaved PARP (poly (ADP-ribose) polymerase-1) (Cell Signaling Technology, Beverly MA, United States), anti-G9a, anti-H3K9me2 (Abcam, Cambridge, United Kingdom), and anti-H3K27me3 (Millipore, Temecula, CA, United States).

### Cell Culture and Treatment With BIX01294 and/or TMZ

Human malignant LN18 and U251 glioma cells were obtained from American Type Culture Collection and were cultured as previously described (Zupanska et al., 2005; Ciechomska et al., 2013). Cell lines were authenticated using Multiplex Cell Authentication by Multiplexion in Heidelberg, Germany. BIX01294 was dissolved in water. TMZ was dissolved in DMSO which was used as a control in corresponding concentrations.

### Sphere Cultures

For sphere forming assay, cells were seeded at a low density (1500 viable cells/cm^2^) onto non-adherent plates and cultured in DMEM/F-12 medium, supplemented with 2% B27 (Gibco Invitrogen, Basel, Switzerland), 20 ng/ml rh bFGF (Miltenyi Biotec, Bergisch Gladbach, Germany), 20 ng/ml rh EGF (StemCell Technologies, Vancouver, BC, Canada), 0.0002% heparin (StemCell Technologies, Vancouver, BC, Canada) and antibiotics (Gibco Invitrogen, Basel, Switzerland). Cells were fed every 3 days by adding 25% of the medium. After 8 days of culturing the spheres were collected by centrifugation at 110 × g and lysed in Qiagen RLT lysis buffer or lysed in buffer supplemented with complete protease inhibitor cocktail (Roche Applied Science, Indianapolis, IN, United States) for blotting, or fixed with 4% paraformaldehyde (PFA) for immunocytochemistry.

### Cell Viability Evaluation by MTT Metabolism or Clonogenic Survival Assay

The cell viability was assayed by measuring the conversion of MTT to formazan (the rate of this reaction is proportional to the number of surviving cells) as previously described (Ciechomska et al., 2005). Briefly, upon cell treatment, MTT stock solution was added at the final concentration 0.5 mg/ml. After 1 h of incubation at 37°C water insoluble formazan was dissolved in lysis buffer containing 20% SDS and 50% DMF. Optical densities were measured at 570 nm using a scanning multi-well spectrophotometer. For clonogenic assay, 48 h after transfection with control siRNA or siRNA targeting G9a cells were seeded as a single cell suspension on 6-well plates (500 or 1000 per well), allowed to adhere for 18 h and then expose to TMZ (500 μM) for 72 h. Subsequently, the medium was replace with drug-free medium, and cells were incubated for 7 days. Plates were stained with 0.1% crystal violet, and colonies were counted by two independent researchers. Results were normalized to the colony-forming efficacy of the cells transfected with control siRNA and DMSO-treated.

### Immunoblotting and Immunofluorescence

Whole cell lysates were prepared by scraping the cells in a buffer containing phosphatase and protease inhibitors, separated by SDS-PAGE and transferred onto nitrocellulose membranes as described (Ciechomska et al., 2005). After blocking with 5% non-fat milk in a blocking buffer the membranes were incubated overnight with primary antibodies and then with relevant secondary antibodies for 1 h. Immunocomplexes were visualized using the enhanced chemiluminescence detection system (SuperSignal West Pico PLUS, Thermo Scietific, United States). The molecular weight of proteins was estimated with pre-stained protein markers (Sigma-Aldrich, Saint Louis, MO, United States).

Adherent cells grown on coverslips were fixed with 4% PFA and then nuclei were counterstained with 1 μg/ml DAPI. For spheres, upon treatments spheres were gently, mechanically dissociated in order to obtain single cell suspension, then cells were cytospined, fixed with PFA and their nuclei were stained with DAPI. The images were acquired with Olympus X70 fluorescent microscope.

### Bisulfite DNA Conversion and Methylation-Specific Polymerase Chain Reaction (MS-PCR)

DNA was extracted using standard phenol/chloroform methods. The purity and concentration of DNA was estimated at the absorbance of 260 and 280 nm. DNA (2 μg) was treated with chemical bisulfite modification (EpiTect Bisulfite Kit, Qiagen, Hilden, Germany) to convert unmethylated, but not the methylated, cytosines to uracil. The modified DNA was then amplified using primers specific for either methylated or unmethylated *MGMT* gene promoter sequences, as listed in Supplementary Table S1. Each PCR mixture contained 1 μl of DNA, 500 nM of primers, 1x reaction buffer containing 1.5 mM MgCl_2,_ 1 U HotStarTaq DNA Polymerase and 250 mM dNTPs (Promega, United States). PCR was performed with thermal conditions as: 95°C for 10 min, 45 cycles of 95°C for 30 s, 57°C for 30 s and 72°C for 30 with a final extension of 72°C for 10 min. The PCR reaction was carried out in two separate tubes specific for methylated and unmethylated sequences. The PCR products were visualized using 1.5% agarose gel or Agilent Bioanalyzer yielding a band of 81 bp for a methylated and 93 bp for an unmethylated product. Positive methylated and positive unmethylated controls (EpiTect PCR Control DNA Set Qiagen, Hilden, Germany) are used to confirm methylation evaluation assays.

### Quantitative RT (Real Time) PCR (qRT-PCR)

Total cellular RNA was extracted using the RNeasy Mini kit (Qiagen, Hilden, Germany) and purified using RNeasy columns according to the manufacturer’s instructions. The integrity of RNA was checked using the Agilent’s 2100 Bioanalyzer. For qRT-PCR total RNA from untreated or BIX01294-treated spheres was used to synthesize cDNA by extension of oligo(dT)_15_ primers with M-MLV reverse transcriptase (Sigma-Aldrich, Munich, Germany). Real-time PCR amplifications were performed in duplicates on cDNA equivalent to 18.75 ng RNA in 10-μl reaction volume containing 2× SYBR GREEN FAST PCR Master Mix (Applied Biosystems, Darmstadt, Germany) and a set of primers. *NANOG* was from Qiagen (QT01844808, Hilden, Germany). Sequences of primers for *CD133* and *SOX2* are in Supplementary Table S1. Data were analyzed by the Relative Quantification method using StepOne Software (Applied Biosystems, Darmstadt, Germany). The expression of each product was normalized to 18S rRNA and is shown as log2 fold changes for all genes.

### Transfection, RNA Interference With siRNA

On-TARGET plus SMART pool siRNA human EHMT2 (G9a) from Dharmacon (Thermo Scientific, Lafayette, CO, United States) was used. Glioma cells were cultured in 6-well plates at a density 3 × 10^5^ cells per well and transfected with 60 nM siRNA. After 48 h, the cells were treated with TMZ (500 μM) for the next 72 h, then harvested and analyzed for cell viability, Western blot. Efficacy of gene knockdown was determined by Western blots and RT-PCR.

### Statistical Analysis

Data were analyzed by Student’s *t*-test or by Newman-Keuls *post-hoc* test (one-way analysis of variance) using Statistica (ver.13.1 StatSoft Inc., Tulsa, OK, United States) software and are presented as mean ± SD or SEM. ^∗^*P* < 0.05, ^∗∗^*P* < 0.01, ^∗∗∗^*P* < 0.001 were considered statistically significant.

## Results

### Anti-Tumor Effect of TMZ and BIX01294 on Human Malignant Glioma Cells

**FIGURE 1:**
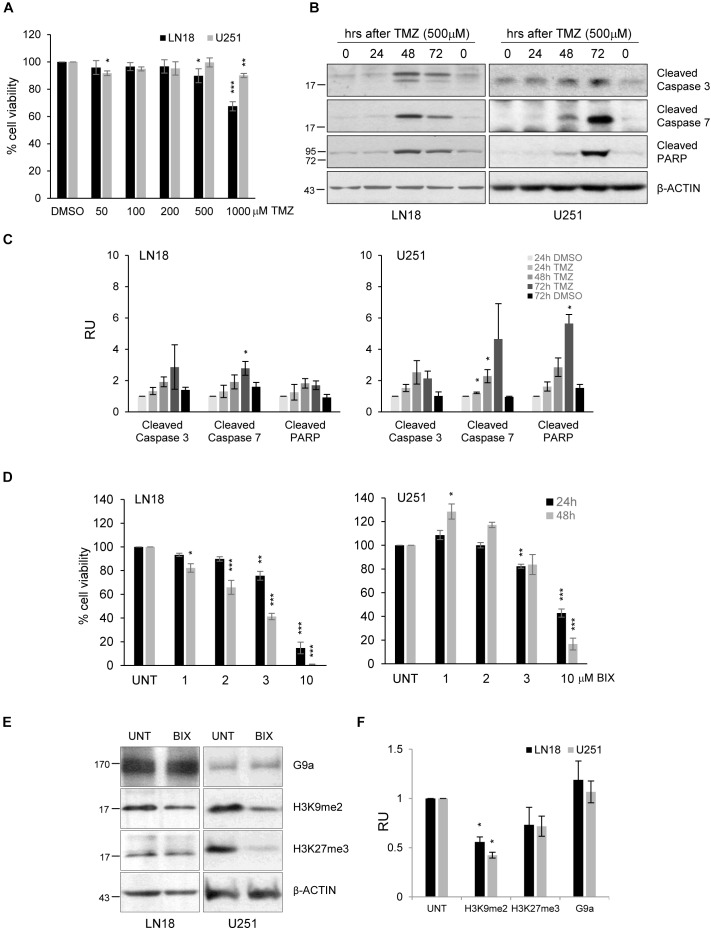
The effects of TMZ or BIX01294 on viability of human glioma cells. **(A)** Cell viability of human LN18 and U251 glioma cells treated for 72 h with TMZ (at concentrations 50–1000 μM) was evaluated using MTT metabolism assay. Results are presented as means ± SD of at least three independent experiments. ^∗^*P* < 0.05, ^∗∗^*P* < 0.01, ^∗∗∗^*P* < 0.001 compared to DMSO-treated cells (*t*-test). **(B)** Western blot analysis of biochemical markers of apoptosis. Immunoblot shows the levels of cleaved caspase 3, caspase 7 and PARP in control, and TMZ-treated glioma cells. Antibody against β-Actin was used to ensure an equal protein loading. **(C)** Densitometric analysis of the blots and quantification of the results from three independent experiments was shown; bars represent means ± SEM of the cleaved caspases, and cleaved PARP levels normalized to β-Actin and then to the control (DMSO-treated cells). **(D)** Cell viability of human LN18 and U251 glioma cells treated to BIX01294 (at concentrations 1–10 μM) was evaluated using MTT metabolism assay. Cells were treated for 24 and 48 h. Results are presented as means ± SD of three independent experiments. ^∗^*P* < 0.05, ^∗∗^*P* < 0.01, ^∗∗∗^*P* < 0.001 compared to untreated control cells (*t*-test). **(E)** LN18 and U251 glioma cells were treated with 2 μM BIX01294 for 48 h. Western blot analysis was performed using the specified antibodies. Levels of G9a, H3K9me2, H3K27me3 proteins were analyzed. Equal protein loading was ensured by β-Actin immunodetection. **(F)** The results of densitometric analysis of the blots obtained in three independent experiments are presented (means ± SEM). The levels of H3K9me2, H3K27me3, and G9a were normalized to β-Actin levels (^∗^*P* < 0.05, *t*-test).

The experiments were performed using human U251 and LN18 glioma cell lines with different status of the *MGMT* gene promoter. MGMT methylation was measured by using methylation specific (MS)-PCR which revealed the unmethylated *MGMT* gene promoter (MGMT active) in LN18 cells, and both methylated and unmethylated *MGMT* promoters in U251 cells (Supplementary Figure S1A). LN18 and U251 glioma cells were exposed to TMZ in increasing concentrations (at range = 50–1000 μM) for 72 h. Viability of LN18 cells was slightly reduced after exposure to 500 or 1000 μM TMZ, up to 87% or 65%, respectively (Figure 1A). TMZ did not affect survival of U251 cells, only the minor effect on cell viability was detected at the highest dose of TMZ. The apoptotic hallmarks were determined in cells exposed to TMZ for 24, 48, and 72 h. Treatment with 500 μM TMZ for 48 and 72 h resulted in accumulation of the cleaved caspase 3, caspase 7 and PARP in U251 and LN18 glioma cells (Figures 1C,D). This shows that methylation of the *MGMT* gene promoter does not predict sensitivity to TMZ.

BIX01294 was added to glioma cell cultures at different concentrations (1-10 μM) for 24 and 48 h, and such treatment reduced cell viability in both cell lines in dose and time-dependent manner. U251 cells were more resistant to BIX01294 exposure than LN18 cells (Figure 1D). At 10 μM BIX01294 reduced viability of U251 and LN18 cells. The levels of H3K9me2 were significantly reduced in LN18 and U251 cells exposed to 2 μM BIX01294 for 48 h, as demonstrated using Western blotting (Figures 1E,F). BIX01294 at concentration 2 μM decreased the H3K9me2 level without inducing cell death as previously shown, therefore such dose was used for further analysis.

### Pre-Treatment or Post-Treatment With BIX01294 Sensitizes Glioma Cells to TMZ

**FIGURE 2:**
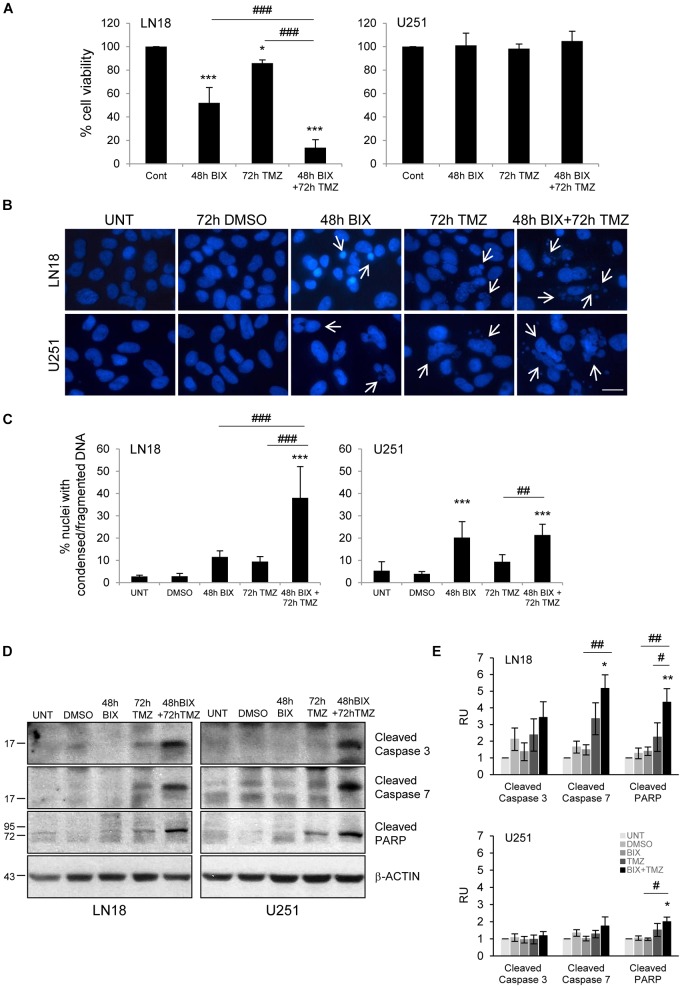
Pre-treatment with BIX01294 enhances the efficacy of TMZ. **(A)** Bar graph shows quantification of cell survival, as measured using MTT assay (mean ± SD, four independent experiments). Cells were treated with each drug alone or pre-treated with BIX01294 (2 μM) for 48 h and were treated for additional 72 h with TMZ (500 μM). One-way analysis of variance followed by Newman-Keuls *post hoc* test; ^∗^*P* < 0.05, ^∗∗∗^*P* < 0.001 compared to control cells (untreated or/and DMSO-treated cells); ^###^P < 0.001 compared to BIX01294 and TMZ-treated cells. **(B)** Representative microphotographs show changes in morphology of the nuclei of cells treated with BIX01294 or TMZ alone or with combination of two drugs. Scale bars represent 20 μm. **(C)** Increased percentages of nuclei with condensed or fragmented DNA in cells exposed to BIX01294 or TMZ or both compounds. Cells with abnormal nuclei were scored. A minimum of 300 cells per sample were counted by two independent researchers (mean ± SD, four independent experiments). ^∗∗∗^*P* < 0.001 compared to untreated control cells. ^##^*P* < 0.01, ^###^*P* < 0.001 BIX01294 or TMZ-treated cells versus cells treated with both drugs (compared using one-way analysis of variance followed by Newman-Keuls *post hoc* test). **(D)** Immunoblot shows the levels of cleaved caspase 3, caspase 7, and PARP in treated glioma cells. Equal protein loading was ensured by β-Actin immunodetection. Note the strongest accumulation of active caspases and cleaved PARP in cells pre-treated with BIX01294 and then treated with TMZ compare to TMZ-treated glioma cells. **(E)** Bar graph showing densitometric evaluation of cleaved caspases and PARP normalized to β-Actin and untreated cells. Each bar represents the mean ± SEM of four independent experiments. ^∗^*P* < 0.05, ^∗∗^*P* < 0.01 compared to untreated control; ^#^*P* < 0.05, ^##^*P* < 0.01 BIX01294, or TMZ-treated cells versus cells treated with both drugs (*post hoc* test in ANOVA).

**FIGURE 3:**
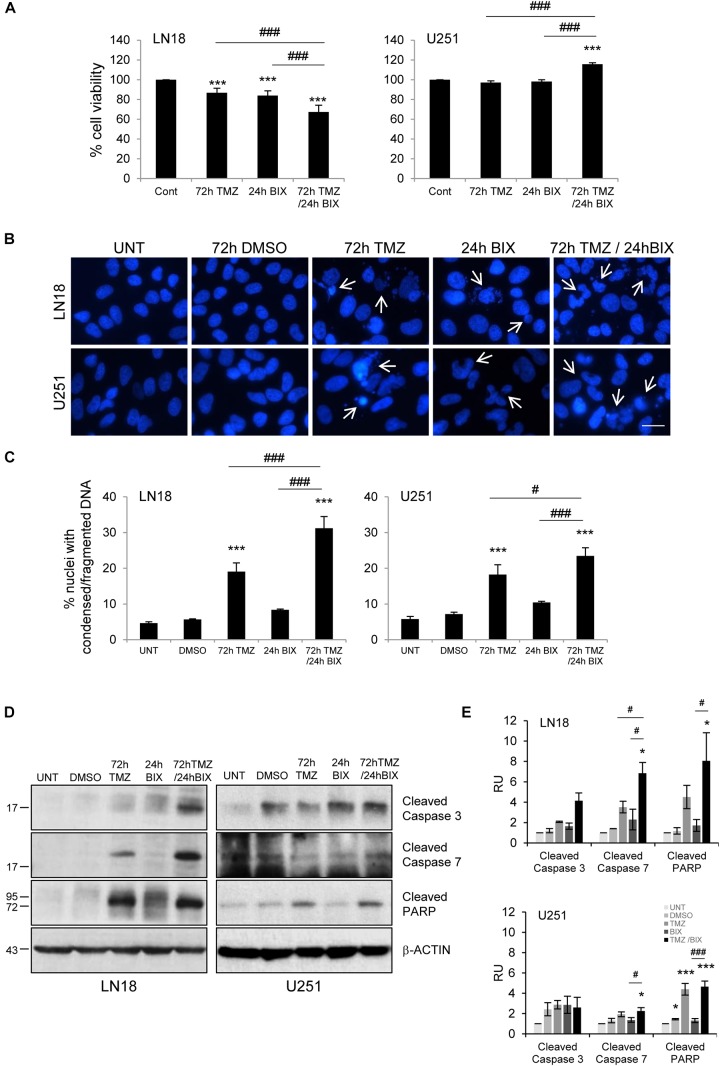
Post-treatment with BIX01294 restores the anti-tumor efficacy of TMZ. **(A)** Bar graph shows quantification of cell survival. Cell viability was determined by MTT metabolism assay. Cells were treated with 500 TMZ μM for 72 h and for the last 24 h cells were treated additionally with 2 μM BIX01294. ^∗∗∗^*P* < 0.001 compared to control cells (untreated or/and DMSO-treated cells); ^###^*P* < 0.001 compared to BIX01294 and TMZ-treated cells (one-way analysis of variance followed by Newman-Keuls *post hoc* test). **(B)** Representative microphotographs show changes in morphology of the nuclei of cells treated with TMZ or BIX01294 alone or with combination of two drugs. Cells were treated first with TMZ followed by BIX01294. Scale bars represent 20 μm. **(C)** Increased percentages of nuclei with condensed or fragmented DNA in cells exposed to TMZ or BIX01294, or both compounds. Cells with abnormal nuclei were scored. A minimum of 300 cells per sample were counted by two independent researchers (mean ± SD, three independent experiments). ^∗∗∗^*P* < 0.001 compared to untreated control cells. ^#^*P* < 0.05, ^###^*P* < 0.001 BIX01294, or TMZ-treated cells versus cells treated with both drugs (*post hoc* test in ANOVA). **(D)** Representative immunoblots of biochemical markers of apoptosis after TMZ or/and BIX01294 incubation in LN18 and U251 glioma cells. Equal protein loading was ensured by β-Actin immunodetection. Similar results were obtained in three independent experiments. **(E)** Bar graph showing densitometric evaluation of cleaved caspases and PARP normalized to β-Actin and untreated cells. Each bar represents the mean ± SEM of three independent experiments. ^∗^*P* < 0.05, ^∗∗∗^*P* < 0.001 compared to untreated control; ^#^*P* < 0.05, ^###^*P* < 0.001 BIX01294, or TMZ-treated cells versus cells treated with both drugs (*post hoc* test in ANOVA).

Since the effect of TMZ alone on glioma cell viability was minor, therefore we tested if combined treatment would be more effective. First, cells were incubated with each compound alone or with BIX01294 for 48 h followed by TMZ for 72 h (pre-treatment) (Figure 2 and Supplementary Figure S2A). In the second set of experiments, cells were first exposed to TMZ for 48 h followed by 24 h co-incubation of BIX01294 and TMZ (post-treatment) (Figure 3 and Supplementary Figure S2B). We analyzed cell viability, changes in morphology of cells and nuclei, and markers of apoptosis such as levels of cleaved caspase 3, caspase 7 and PARP. As shown in Figures 2A, 3A, combination of BIX01294 and TMZ had an additive, anti-tumor effect in LN18 cells, as determined by MTT assay (when combination treatment versus a single treatment were compared). The effect was stronger in the pre-treatment scenario. In contrast, U251 glioma cells were resistant to either treatment. Consistently, the changes in cell morphology induced by BIX01294 or TMZ alone versus drug combination were in agreement with cell viability results (Supplementary Figures S2A,B). Changes in cell morphology appeared in BIX01294-treated LN18 cells 48 h after treatment. Majority of LN18 cells treated with drug combination lost processes and became round, the strongest effect was observed in cultures pre-incubated with BIX01294. Again, U251 cells did not show apparent alterations.

To study if BIX01294 sensitizes to TMZ-induced cell death, we performed an analysis of alterations in morphology of cell nuclei by counting altered/fragmented nuclei stained with DAPI. Control cells had large and round nuclei, and each drug alone produced a small increase in the percentage of deformed nuclei with fragmented/condensed chromatin. After combined BIX01294 and TMZ treatment the number of cells with abnormal apoptotic nuclei significantly increased in LN18 cells and in a lesser extent in U251 cells (Figures 2B,C, 3B,C). Such synergistic changes occurred in LN18 cells irrespectively of the order of drug administration (8.5–11% for BIX01294, 9.5–19% for TMZ versus 31–39% for BIX01294 + TMZ). The synergistic effect of both compounds was not so obvious in U251 cells, although sensitization by BIX01294 to TMZ was observed.

Western blot analysis of cleaved caspases and cleaved PARP shows the increased levels of apoptotic markers in LN18 cells treated with both drugs (Figures 2D,E, 3D,E). PARP cleavage increased significantly also in U251 cells exposed to drug combination. These data suggest that inhibition of histone methyltransferase G9a improves anti-tumor efficacy of TMZ in glioma cells, although the strongest effect was found in LN18 cells. Both pre-treatment or post-treatment with BIX01294 sensitizes glioma cells to TMZ.

### BIX01294 Enhances the Cytotoxic Effect of TMZ on Glioma Stem-Like Cells

**FIGURE 4:**
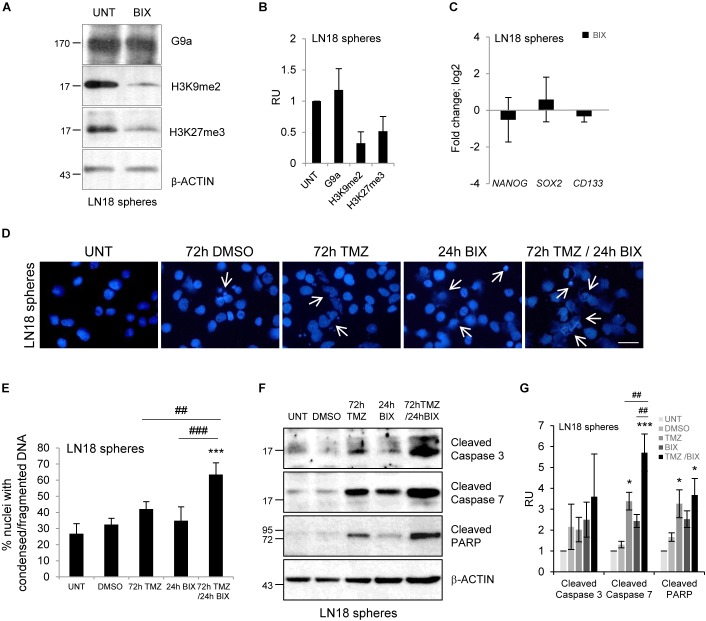
BIX01294 enhances cytotoxic effects of TMZ on glioma spheres. **(A)** Representative immunoblots show G9a, H3K9me2 and H3K27me3 levels in LN18 spheres treated with 2 μM BIX01294 for 24 h. **(B)** Densitometric analysis was performed; bars show means ± SEM of G9a, H3K9me2, and H3K27me3 levels normalized to β-Actin and control (untreated spheres) (*n* = 3). **(C)** Analysis of *NANOG, SOX2*, and *CD133* gene expression by qPCR in LN18 spheres treated with BIX01294 as compared to the untreated spheres (*n* = 6). **(D)** Microphotographs show changes in morphology of the nuclei of LN18 spheres treated with 500 μM TMZ for 72 h or 2 μM BIX01294 for 24 h alone or in combination with two drugs; TMZ was applied prior to BIX01294. Scale bars represent 20 μm. **(E)** Increased percentages of the cells forming spheres with abnormal nuclei after treatment with TMZ or BIX01294 alone, or treated with both compounds sequentially. Cells with abnormal nuclei were scored. A minimum of 300 cells per sample were counted (mean ± SD, three independent experiments). ^∗∗∗^*P* < 0.001 compared to untreated control cells. ^##^*P* < 0.01, ^###^*P* < 0.001 BIX01294 or TMZ-treated cells versus cells treated with both drugs (*post hoc* test in ANOVA). **(F)** Representative immunoblots of biochemical markers of apoptosis after treatment of LN18 spheres with TMZ or/and BIX01294. Note the increased levels of cleaved caspase 3, caspase 7, and PARP in spheres treated with TMZ alone or in combination with BIX01294. Equal protein loading was ensured by β-Actin immunodetection. Similar results were obtained in three independent experiments. **(G)** Bar graph shows densitometric evaluation of cleaved caspases and PARP normalized to β-Actin levels and untreated spheres. Each bar represents the mean ± SEM of three independent experiments. ^∗^*P* < 0.05, ^∗∗∗^*P* < 0.001 compared to untreated control, ^##^*P* < 0.01 BIX01294, or TMZ-treated cells versus cells treated with both drugs (compared using *post hoc* test in ANOVA).

Glioma stem-like cells (GSCs) have numerous mechanisms of resistance to radio- and chemotherapy (Bao et al., 2006; Chen et al., 2012). We explored if BIX01294 would affect the stemness features of GSCs and if it would sensitize glioma cells to TMZ. LN18 cultures enriched in GSCs were obtained by culturing LN18 cells in suspension in the serum-free medium containing growth factors EGF (epidermal growth factor) and bFGF (basic fibroblast growth factor). After 8 days of culture under such conditions LN18 formed tumor spheres of a size c.a. 100 μm which were visualized by light microscopy (Supplementary Figure S3B). GSCs-enriched spheres expressed higher levels of the pluripotency markers: *NANOG, SOX2*, and *CD133* as compared to the parental/adherent tumor cells (Supplementary Figure S3A). BIX01294 reduced levels of H3K9me2 and H3K27me3 in LN18 spheres (Figures 4A,B), however, did not affect the expression of pluripotency genes (Figure 4C). Consistently, BIX01294 treatment did not change DNA methylation at the *NANOG* and *SOX2* genes either in LN18 spheres or in parental cells (Supplementary Figure S3C). TMZ alone induced some apoptotic features such as the increase in percentages of the cells with abnormal nuclei and activation of caspases when compared with their levels in control spheres (Figures 4D,E). However, cells with alterations/fragmentation of nuclei were more numerous when BIX01294 was applied after treatment with TMZ (Figures 4D,E). Consistently treatment with TMZ followed by BIX01294 increased the levels of active caspases and cleaved PARP (Figures 4F,G). These results suggest that post-treatment with BIX01294 enhances the anti-tumor effect of TMZ on LN18 GSCs.

### Knockdown of Methyltransferase G9a Augments TMZ-Induced Apoptotic Cell Death

**FIGURE 5:**
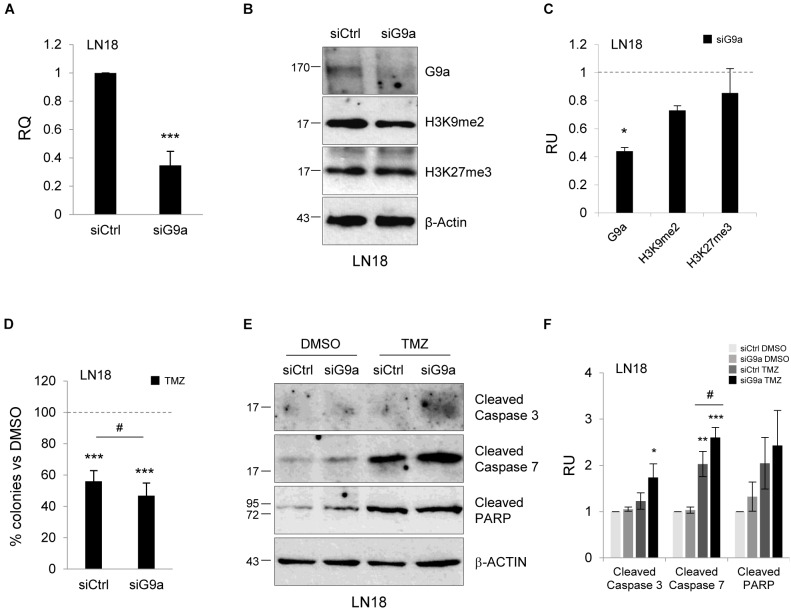
Knockdown of methyltransferase G9a augments TMZ-induced cell death in malignant glioma cells. Efficacy of knockdown of G9a using specific siRNA at the mRNA **(A)** and protein level **(B)** is demonstrated by Westren blotting and qPCR, respectively. Results of three independent experiments are shown (^∗∗∗^*P* < 0.001 compared with scrambled siRNA-transfected cells (siCtrl). Levels of G9a, H3K9me2, H3K27me3 proteins were analyzed. Equal protein loading was ensured by β-Actin immunodetection. **(C)** The results of densitometry analysis of the blots (*n* = 3, means ± SEM). The levels of H3K9me2, H3K27me3 and G9a were normalized to siCtrl-transfected cells and β-Actin levels (^∗^*P* < 0.05, *t*-test). **(D)** Knockdown of G9a affects colony formation in TMZ-treated LN18 cells. Glioma cells were transfected with siCtrl or siG9a, exposed to DMSO or TMZ for 72 h and cell viability was determined by clonogenic survival assay (means ± SD, *n* = 4, ^∗∗∗^*P* < 0.001, compared to DMSO-treated cells, ^#^*P* < 0.05 siCtrl-transfected, TMZ-treated cells versus siG9a-transfected TMZ-treated cells, *post hoc* test in ANOVA) **(E)** Immunoblot show the levels of cleaved caspase 3, 7, and PARP in glioma cells. Note the increase of apoptosis hallmarks in TMZ-treated LN18 glioma cells after inhibition of G9a. **(F)** Densitometry analysis of immunoblots shown in **(E)**. Each bar represents the means ± SEM of three independent experiments.

In addition to pharmacological studies, we performed knockdown of methyltransferase G9a using a gene specific siRNA (a mixture of three targeting siRNAs). We observed effective (60%) silencing of G9a at the mRNA and protein levels (Figures 5A–C). The epigenetic marks, H3K9me2 and H3K27me3, were reduced after knockdown of the G9a expression. To test whether inhibition of G9a contributes to TMZ-induced death, we performed clonogenic assay after knockdown of G9a followed by treatment with TMZ. Inhibition of methyltransferase G9a increased the efficacy of TMZ in reducing colony fomation (Figure 5D). The treatment of LN18 cells with TMZ induced caspase 7 and PARP cleavage (when compared to DMSO-treated cells) and downregulation of G9a expression augmented these events (Figures 5E,F). As described above, we found that genetic inhibition of histone methyltransferase G9a enhanced the TMZ-induced cell death.

### Induction of Autophagy in Glioma Cells Exposed to TMZ and BIX01294

We have previously demonstrated that BIX01294 induces autophagy or autophagy-associated cell death in glioma cell cultures and GSCs (Ciechomska et al., 2016). We explored if autophagy plays a role in BIX01294-induced sensitization to TMZ. Upon autophagy induction, LC3-I is converted to LC3*-*II, which correlates with an increase in autophagic vacuoles (Kabeya et al., 2004), therefore we analyzed the level of LC3 in treated cell cultures. We found the significant increase of the LC3-II level in LN18 cells upon BIX01294 treatment and the slightly stronger effect in cells treated with TMZ and BIX01294 (Supplementary Figures S4A,B). No such changes occurred in U251 cells exposed to drug combination or each drug alone. If autophagy-associated cell death is involved in TMZ induced killing of those cells, its lack could explain a weak effect of drugs on U251 cells.

As H3K9me2 could participate in the *MGMT* gene silencing (Zhao et al., 2005), we investigated whether the *MGMT* promoter status was affected by treatment of cells with BIX01294. MS-PCR assay revealed that BIX01294 treatment does not affect *MGMT* status, as the *MGMT* promoter remains unmethylated in treated cells (Supplementary Figure S1B). This result suggests that sensitization to TMZ is not associated with the changes of the *MGMT* status in LN18 cells.

## Discussion

Epigenetic drugs can restore the epigenetic landscape in cancer cells by inhibiting enzymes carrying on epigenetic modifications (Kouzarides, 2007). G9a, an enzyme responsible for histone H3 lysine 9 (H3K9) mono- and dimethylation, is upregulated in different cancers. We investigated if G9a inhibitor will sensitize glioma cells to TMZ, commonly used in anti-glioma therapy. As responsiveness to TMZ differs in various cells, we used two glioma cell lines known to differ in sensitivity to TMZ due to the presence or absence of the MGMT protein (Lee, 2016). Although, we found differences in the *MGMT* promoter status in LN18 and U251 cells (with U251 cells having both methylated and unmethylated *MGMT*), there was no correlation with responses to TMZ (Figures 1A–C and Supplementary Figure S1A). Surprisingly, U251 glioma cells were more resistant to TMZ than LN18 and TMZ at the highest dose only slightly reduced viability of U251 cells. The searches indicated that TMZ induced cytotoxicity in U251 cells varies and occurs at the range from <20 μM till 500 μM (Lee, 2016). We explored if epigenetic reprograming could sensitize cells to TMZ and restore their capacity to undergo apoptosis.

We targeted histone methyltransferase G9a (by pharmacological inhibition or genetic ablation of G9a) which is upregulated in many cancers and implicated in cancerogenesis (Casciello et al., 2015). BIX01294 (a competitive inhibitor specific for G9a) was administrated before or after adding TMZ. We show that BIX01294 reduced H3K9me2 level in both LN18 and U251 cell lines, as well as in LN18 GSCs (Figures 1E,F, 4A), which is in agreement with previous studies (Maleszewska et al., 2014; Ciechomska et al., 2016). We demonstrate for the first time that 2 μM BIX01294 (a dose non-inducing cell death) (Ciechomska et al., 2016) improves anti-tumor efficacy of TMZ in human glioma cells. Pre-treatment, as well as post-treatment enhanced cytotoxicity of TMZ and restored cell ability to undergo apoptosis, as was evidenced by reduced cell viability, the increased number of cells with abnormal/fragmented nuclei and activation of a caspase cascade (Figures 2, 3). Although, sensitization to TMZ varied among glioma cell lines. Differences in the response of LN18 and U251 cells could be explained by differences in their genetic background (Ishii et al., 1999). The synergistic effect of both compounds was mostly observed in LN18 cells. Furthermore, silencing of G9a expression significantly increased the efficacy of TMZ as demonstrated by reduced colony fomation, and increased the level of active caspases in TMZ-treated LN18 cells (Figure 5). This is in agreement with the results showing that G9a knockdown inhibit the proliferation and survival of cancer cell lines (Chen et al., 2010; Huang et al., 2010; Ding et al., 2013; Wojtala et al., 2018). The molecular basis of G9a action in the control of cancer cell proliferation is not well understood. Some data suggest that G9a sustains cancer cell survival and proliferation by the control of amino acid production, cancer metabolism (Ding et al., 2013) or by the regulation genes expression involved in the cell cycle machinery (Wojtala et al., 2018).

Glioma stem-like cells maintain tumor growth, drive tumor progression and cause tumor relapse due to their increased resistance to therapies (Reya et al., 2001; Bao et al., 2006; Chen et al., 2012). Interestingly, we present evidence suggesting that BIX01294 enhanced the cytotoxic effect of TMZ on glioma stem-like cells (Figures 4D–G). We expanded GSCs from glioma LN18 cells in serum-free medium with growth factors and confirmed the higher expression of pluripotency markers (*NANOG, SOX2, CD133*) in GSCs-enriched spheres, as previously shown (Ciechomska et al., 2016; Kijewska et al., 2017). However, BIX01294 did not affect the expression and methylation status of stemness genes (Figure 4C and Supplementary Figure S3C). The effects of BIX01294 on CSCs are conflicting. In previous studies BIX01294 was shown to stimulate sphere formation and increase *SOX2* and *CD133* expression in CD133-positive glioma stem cells (Tao et al., 2014; Kim et al., 2017). We previously found that BIX01294 induces up-regulation of GFAP (glial fibrillary acidic protein) and β-Tubulin that are differentiation markers in LN18 GSCs and GBM patient derived GSCs. BIX01294-induced differentiation was dependent on the transcriptional activation of autophagy genes (Ciechomska et al., 2016). Induction of autophagy or autophagy-associated cell death upon BIX01294 or TMZ treatment in glioma cells was observed (Kanzawa et al., 2004; Ciechomska et al., 2016). In this study, we observed accumulation of LC3-II (an autophagy marker) in LN18 upon BIX01294 alone or TMZ and BIX01294 treatment. BIX01294 slightly upregulated the LC3-II level in U251 cells (Supplementary Figures S4A,B). LN18 and U251 are characterized by wild-type and mutated PTEN (phosphatase and tensin homolog), respectively (Ishii et al., 1999), which may alter pathways activating autophagy (Liang and Jung, 2010). We investigated whether BIX01294 influences on the methylation status of *MGMT* promoter reflecting MGMT expression. Apparently, BIX01294 treatment did not affect methylation status of the *MGMT* gene promoter (Supplementary Figure S1B). The mechanisms underlying BIX01294 induced reprograming and sensitization of glioma cells to TMZ require further elucidation. Characterization of G9a target genes by whole transcriptome analysis in untreated and BIX01294-treated glioma cells may provide better understanding of a role of epigenetic reprograming which regulates drug sensitivity and cell ability to undergo apoptosis. Several studies have shown that targeting epigenetic enzymes with valproic acid (VPA) or suberoylanilide hydroxamic acid (SAHA) potentiated efficacy of TMZ in glioma cells (Das et al., 2007; Ryu et al., 2012; Van Nifterik et al., 2012; Diss et al., 2014).

To conclude, in the present study we demonstrated that BIX01294 (the inhibitor of G9a) sensitizes selected glioma cells to TMZ. A number of studies have shown an important role for G9a in progression of solid tumors and metastasis (Casciello et al., 2015), therefore we suggest that G9a is a potential therapeutic target in malignant gliomas. Our results suggest that G9a inhibitor can be used alone or in combination with other standard therapies (i.e., TMZ) to achieve better anti-tumor effects in GBM patients. Nowadays, inhibitors of histone methyltransferases have not yet reached the clinical trial stage, however, various HDAC inhibitors, including valproic acid have been approved by the FDA and are tested against different types of malignancies, including glioblastoma (Weller et al., 2011; Berendsen et al., 2012; Krauze et al., 2015).

## Author Contributions

IAC and BK designed the study, interpreted data and wrote the manuscript. IAC, MPM and JJ performed experiments. All the authors read the manuscript.

## Conflict of Interest Statement

The authors declare that the research was conducted in the absence of any commercial or financial relationships that could be construed as a potential conflict of interest.

## Supplementary Material

FIGURE S1 The methylation status of the *MGMT* gene promoter in LN18 and U251 glioma cells. **(A)** *MGMT* gene promoter methylation status in LN18 and U251 glioma cells was determined by methylation-specific PCR assay and visualized using Agilent Bioanalyzer. The methylated, as well as unmethylated, bisulfite converted DNA were used as a positive controls (PC) for methylated (M) or unmethylated (U) templates, respectively. NC, negative control for methylated and unmethylated DNA. H_2_0, control without DNA. A 100-bp marker ladder was loaded to estimate molecular size (right). Representative results of two independent experiments were shown. **(B)** *MGMT* gene promoter methylation status in control and BIX01294-treated LN18 cells was determined using methylation-specific PCR assay. The PCR products were separated on 1.5% agarose gel, visualized by SimplySafe staining. PC, positive controls for methylated or unmethylated DNA, respectively. NC, negative control for methylated and unmethylated DNA. H_2_0, control without DNA.

FIGURE S2 Combination of BIX01294/TMZ induced morphological changes in glioma cells. Schematic representation of the treatment protocols. Cells were incubated with BIX01294 for 48 h before adding TMZ for 72 h (pre-treatment) (**A**, upper panel) or 48 h after expose to TMZ followed by 24 h co-incubation of BIX01294 and TMZ (post-treatment) (**B**, upper panel). Representative microphotographs show morphology changes of LN18 and U251 glioma cells treated with BIX01294 or TMZ alone or with combination of two drugs. Changes in cell morphology were monitored by phase-contrast microscopy. (**A**, lower panel) Pictures were taken after 48 h of BIX01294 (2 μM) treatment and/or additional 72 h with TMZ (500 μM). Scale bars represent 50 μm. (**B**, lower panel) Pictures were taken after 72 h of TMZ (500 μM) treatment or 24 h of BIX1294 (2 μM) treatment alone. Additionally, TMZ was treated for 48 h prior to BIX01294, which was added for additional 24 h together with TMZ. Scale bars represent 50 μm.

FIGURE S3 Combining BIX01294 and TMZ induced morphological changes in glioma stem-like cells. **(A)** Quantitative analysis of *NANOG, SOX2* and *CD133* gene expression in LN18 neurospheres (growing in the serum-free medium containing rh EGF and rh bFGF) as compared to the parental/adherent cells (growing in the presence of serum) (*n* = 6, ^∗∗^*P* < 0.01, ^∗∗∗^*P* < 0.001, *t*-test). **(B)** Photographs show changes in morphology of LN18 spheres after adding BIX01294 for 24 h, TMZ for 72 h or treated with both compounds sequentially. TMZ was treated prior to BIX01294. Scale bars represent 100 μm. **(C)** *NANOG* and *SOX2* gene promoter methylation in control and BIX01294-treated adherent LN18 and LN18 spheres was determined using methylation-specific PCR assay. The PCR products were separated on 1.5% agarose gel, visualized by SimplySafe staining. PC, positive controls for methylated or unmethylated DNA, respectively. NC, negative control for methylated and unmethylated DNA. H_2_0, control without DNA.

FIGURE S4 Induction of autophagy in glioma cells by BIX01294 and TMZ combination. **(A)** Conversion of LC3-I to LC3-II was determined by Western blotting. β-Actin was used as a loading control. LN18 cells were exposed to 2 μM BIX01294 for 48 h or 500 μM TMZ for 72 h alone or in combination with two drugs. Treatment with BIX01294 preceded a treatment with TMZ. The results are representative of four independent experiments. **(B)** Bar graph shows densitometric evaluation of the ratio of LC3-II/LC3-I normalized to β-Actin levels and untreated cells. Each bar represents the mean ± SEM of four independent experiments. ^∗^*P* < 0.05, ^∗∗^*P* < 0.01 compared to untreated control. ^#^*P* < 0.05 BIX01294 or TMZ-treated cells versus cells treated with both drugs (*post hoc* test in ANOVA).

TABLE S1 Sequences of primers used in this work.
