# Supplementary figures and images for: Pre-treatment or Post-treatment of Human Glioma Cells With BIX01294, the Inhibitor of Histone Methyltransferase G9a, Sensitizes Cells to Temozolomide
Source: Front Pharmacol. 2018 Nov 2;9:1271. doi: 10.3389/fphar.2018.01271 (PMC6224489; doi:10.3389/fphar.2018.01271)

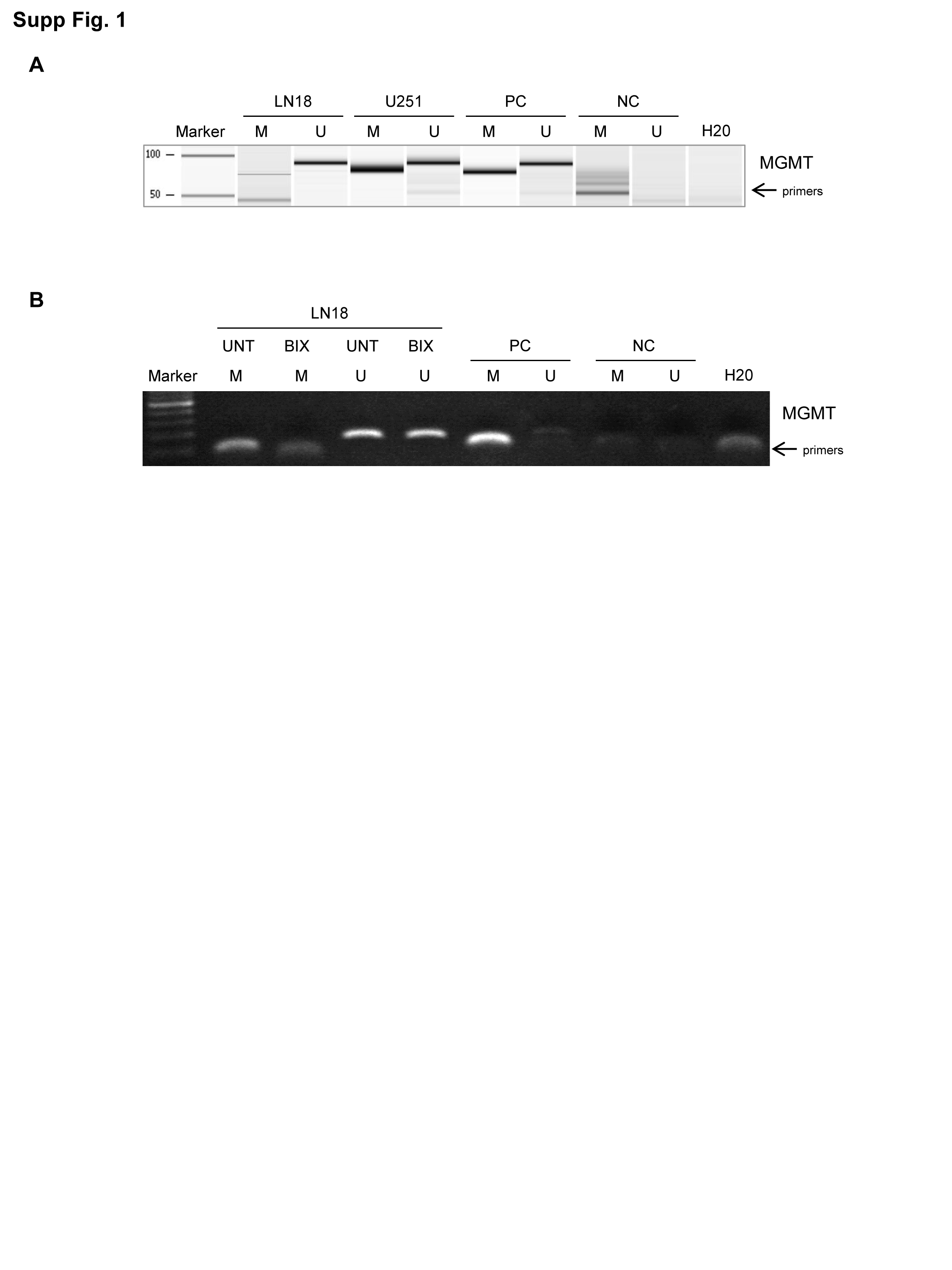

Supplement: FIGURE S1 — The methylation status of the MGMT gene promoter in LN18 and U251 glioma cells. (A) MGMT gene promoter methylation status in LN18 and U251 glioma cells was determined by methylation-specific PCR assay and visualized using Agilent Bioanalyzer. The methylated, as well as unmethylated, bisulfite converted DNA were used as a positive controls (PC) for methylated (M) or unmethylated (U) templates, respectively. NC, negative control for methylated and unmethylated DNA. H20, control without DNA. A 100-bp marker ladder was loaded to estimate molecular size (right). Representative results of two independent experiments were shown. (B) MGMT gene promoter methylation status in control and BIX01294-treated LN18 cells was determined using methylation-specific PCR assay. The PCR products were separated on 1.5% agarose gel, visualized by SimplySafe staining. PC, positive controls for methylated or unmethylated DNA, respectively. NC, negative control for methylated and unmethylated DNA. H20, control without DNA. [file Image_1.TIF]

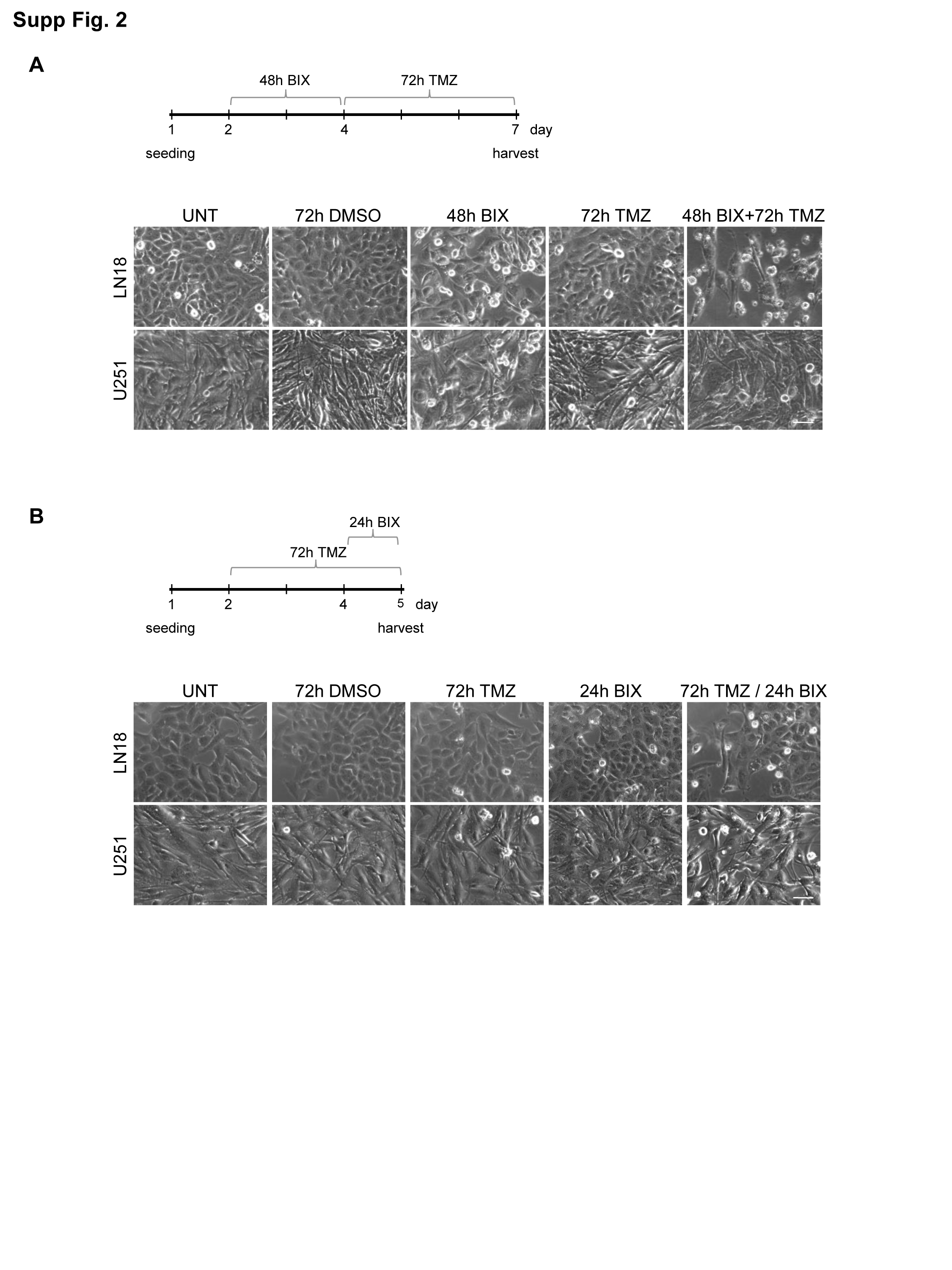

Supplement: FIGURE S2 — Combination of BIX01294/TMZ induced morphological changes in glioma cells. Schematic representation of the treatment protocols. Cells were incubated with BIX01294 for 48 h before adding TMZ for 72 h (pre-treatment) (A, upper panel) or 48 h after expose to TMZ followed by 24 h co-incubation of BIX01294 and TMZ (post-treatment) (B, upper panel). Representative microphotographs show morphology changes of LN18 and U251 glioma cells treated with BIX01294 or TMZ alone or with combination of two drugs. Changes in cell morphology were monitored by phase-contrast microscopy. (A, lower panel) Pictures were taken after 48 h of BIX01294 (2 μM) treatment and/or additional 72 h with TMZ (500 μM). Scale bars represent 50 μm. (B, lower panel) Pictures were taken after 72 h of TMZ (500 μM) treatment or 24 h of BIX1294 (2 μM) treatment alone. Additionally, TMZ was treated for 48 h prior to BIX01294, which was added for additional 24 h together with TMZ. Scale bars represent 50 μm. [file Image_2.TIF]

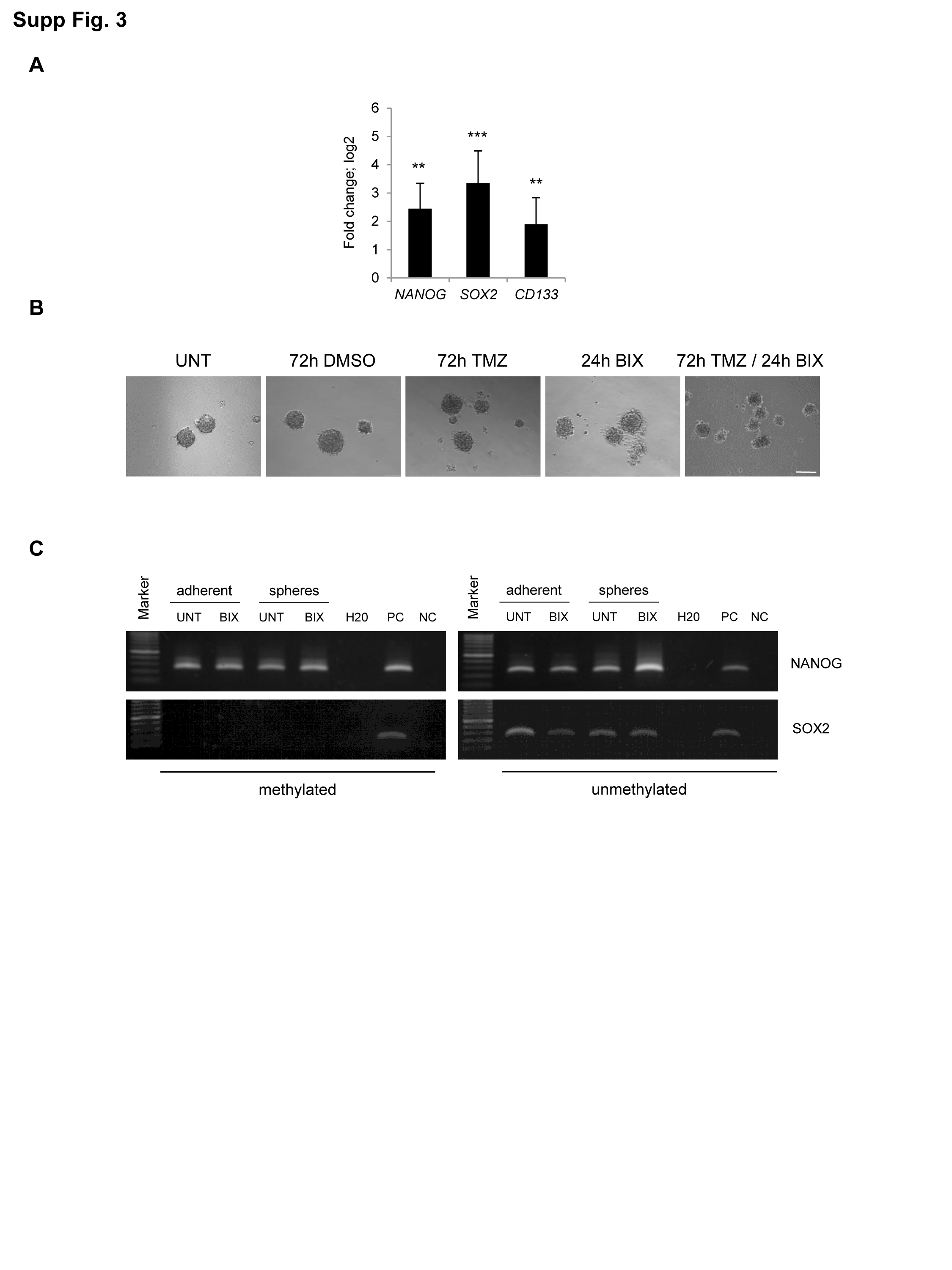

Supplement: FIGURE S3 — Combining BIX01294 and TMZ induced morphological changes in glioma stem-like cells. (A) Quantitative analysis of NANOG, SOX2 and CD133 gene expression in LN18 neurospheres (growing in the serum-free medium containing rh EGF and rh bFGF) as compared to the parental/adherent cells (growing in the presence of serum) (n = 6, ∗∗P < 0.01, ∗∗∗P < 0.001, t-test). (B) Photographs show changes in morphology of LN18 spheres after adding BIX01294 for 24 h, TMZ for 72 h or treated with both compounds sequentially. TMZ was treated prior to BIX01294. Scale bars represent 100 μm. (C) NANOG and SOX2 gene promoter methylation in control and BIX01294-treated adherent LN18 and LN18 spheres was determined using methylation-specific PCR assay. The PCR products were separated on 1.5% agarose gel, visualized by SimplySafe staining. PC, positive controls for methylated or unmethylated DNA, respectively. NC, negative control for methylated and unmethylated DNA. H20, control without DNA. [file Image_3.TIF]

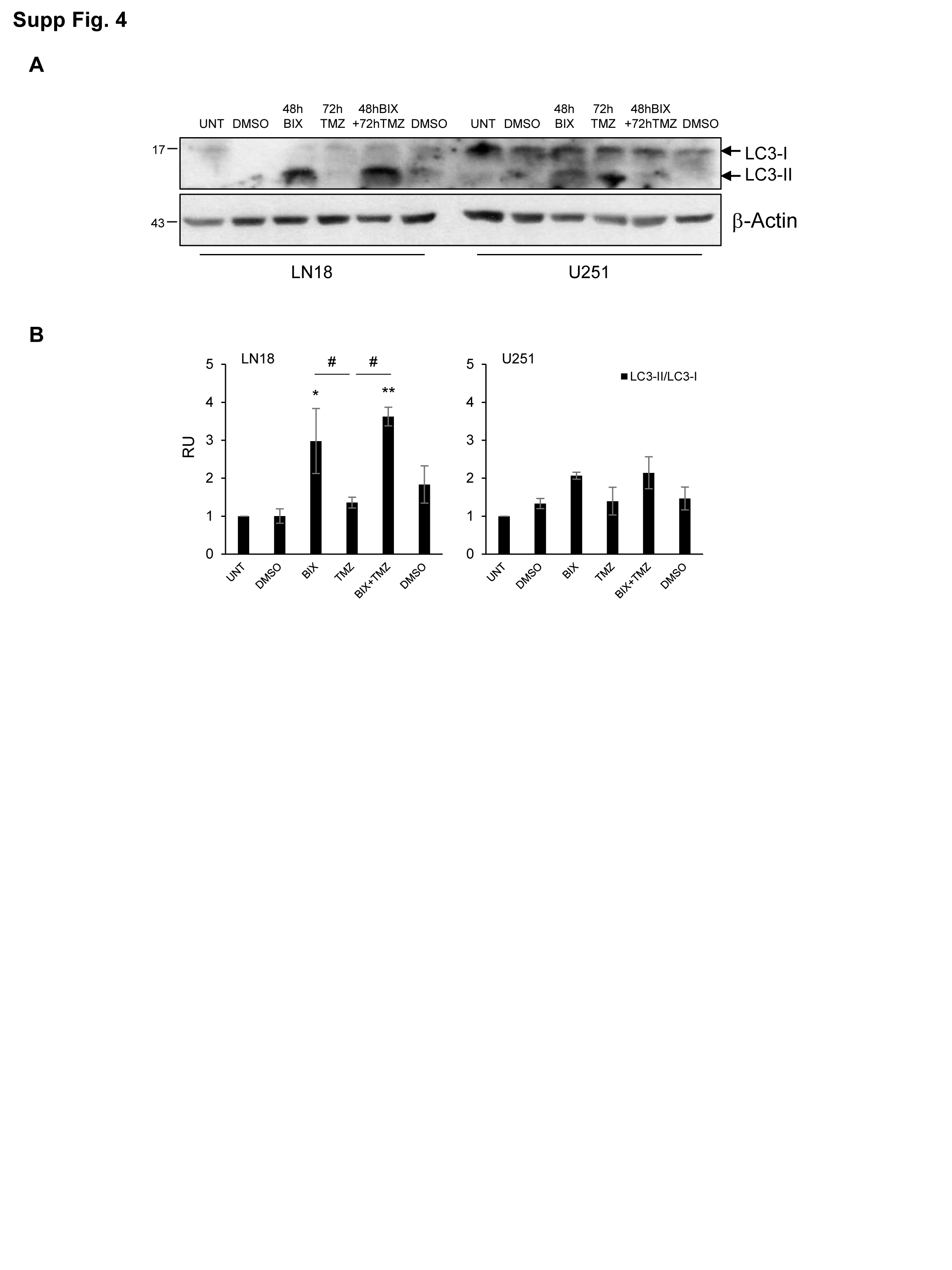

Supplement: FIGURE S4 — Induction of autophagy in glioma cells by BIX01294 and TMZ combination. (A) Conversion of LC3-I to LC3-II was determined by Western blotting. β-Actin was used as a loading control. LN18 cells were exposed to 2 μM BIX01294 for 48 h or 500 μM TMZ for 72 h alone or in combination with two drugs. Treatment with BIX01294 preceded a treatment with TMZ. The results are representative of four independent experiments. (B) Bar graph shows densitometric evaluation of the ratio of LC3-II/LC3-I normalized to β-Actin levels and untreated cells. Each bar represents the mean ± SEM of four independent experiments. ∗P < 0.05, ∗∗P < 0.01 compared to untreated control. #P < 0.05 BIX01294 or TMZ-treated cells versus cells treated with both drugs (post hoc test in ANOVA). [file Image_4.TIF]
