# Supplementary material for: Pre-treatment or Post-treatment of Human Glioma Cells With BIX01294, the Inhibitor of Histone Methyltransferase G9a, Sensitizes Cells to Temozolomide
Source: Front Pharmacol. 2018 Nov 2;9:1271. doi: 10.3389/fphar.2018.01271 (PMC6224489; doi:10.3389/fphar.2018.01271)
Supplement: TABLE S1 — Sequences of primers used in this work. [file Table_1.docx]

**Supplementary Table S1**

Sequences of primers used in this work

| SOX2_qPCR | GGGGAAAGTAGTTTGCTGCC | CGCCGCCGATGATTGTTATT |
| --- | --- | --- |
| CD133_qPCR | TGGATGCAGAACTTGACAACGT | ATACCTGCTACGACAGTCGTGGT |
| 18S_qPCR | CGGACATCTAAGGGCATCAC | AACGAACGAGACTCTGGCAT |
| met MGMT | TTTCGACGTTCGTAGGTTTTCGC | GCACTCTTCCGAAAACGAAACG |
| unmet MGMT | TTTGTGTTTTGATGTTTGTAGGTTTTTGT | AACTCCACACTCTTCCAAAAACAAAACA |
| met NANOG | AATTAAAAAAAAAACCGAACGC | TAGGTTGGAGTATAGTGGCGC |
| unmet NANOG | AAAAATTAAAAAAAAAACCAAACAC | GTTTAGGTTGGAGTATAGTGGTGT |
| met SOX2 | AACCTCGCTACCGAATTTT | TTTATTTATTTTTTTCGAAAAGGC |
| unmet SOX2 | TTTCTAACAACCAATCAACACACA | TTGTTTATTTATTTTTTTTGAAAAGGT |
